# Engineering Sub-Cellular Targeting Strategies to Enhance Safe Cytosolic Silica Particle Dissolution in Cells

**DOI:** 10.3390/pharmaceutics12060487

**Published:** 2020-05-28

**Authors:** Nerea Iturrioz-Rodríguez, Miguel Ángel Correa-Duarte, Rafael Valiente, Mónica L. Fanarraga

**Affiliations:** 1 Grupo de Nanomedicina, Instituto Valdecilla-IDIVAL, Herrera Oria s/n, 39011 Santander, Spain; rafael.valiente@unican.es; 2 Biomedical Research Centre (CINBIO), Universidade de Vigo, 36310 Vigo, Spain; macorrea@uvigo.es; 3 Southern Galicia Institute of Health Research (IISGS), and CIBERSAM, 36213 Vigo, Spain; 4 Departments of Applied Physics, University of Cantabria, 39005 Santander, Spain; 5 Molecular Biology, University of Cantabria, 39011 Santander, Spain

**Keywords:** silica nanocarrier, cytoplasmic escape, biodegradation, engineering nanoparticles, HeLa, motor neurons

## Abstract

Mesoporous silica particles (MSP) are major candidates for drug delivery systems due to their versatile, safe, and controllable nature. Understanding their intracellular route and biodegradation process is a challenge, especially when considering their use in neuronal repair. Here, we characterize the spatiotemporal intracellular destination and degradation pathways of MSP upon endocytosis by HeLa cells and NSC-34 motor neurons using confocal and electron microscopy imaging together with inductively-coupled plasma optical emission spectroscopy analysis. We demonstrate how MSP are captured by receptor-mediated endocytosis and are temporarily stored in endo-lysosomes before being finally exocytosed. We also illustrate how particles are often re-endocytosed after undergoing surface erosion extracellularly. On the other hand, silica particles engineered to target the cytosol with a carbon nanotube coating, are safely dissolved intracellularly in a time scale of hours. These studies provide fundamental clues for programming the sub-cellular fate of MSP and reveal critical aspects to improve delivery strategies and to favor MSP safe elimination. We also demonstrate how the cytosol is significantly more corrosive than lysosomes for MSP and show how their biodegradation is fully biocompatible, thus, validating their use as nanocarriers for nervous system cells, including motor neurons.

## 1. Introduction

The success of nanotechnology applied in health requires the use of materials with well-defined in vivo functionalities. Thus, understanding the biocompatibility, intracellular destination, biodegradation and elimination routes of nanomaterials are critical issues. 

Nanomaterial biodegradation is not the main issue in cancer treatment. The high proliferation rate of the tumoral and peritumoral cells results in a progressive reduction of the nanomaterial loaded per cell to tolerable amounts [1,2,3]. On the contrary, if the nanodevices are designed for cell repair, for instance, destined to lower proliferative cells such as neurons or their supporting glial cells, they are likely to accumulate intracellularly or extracellularly, triggering unpredictable negative side-effects [4]. 

Besides, identifying the cellular compartment where nanomaterials are degraded and accumulate can also serve to control/predict spatiotemporally the release of the therapeutic compound and to customize nanoparticles ad hoc, preserving some therapeutic agents from early degradation in hostile cellular environments, i.e., the lysosomes [5,6]. Regrettably, there are no standards for a fully comprehensive and reliable characterization of the diversity and complexity of nanomaterials, or protocols to predict the interaction of these or their degradation products, with proteins, cells, and tissues [7].

Recent advances in nanotechnology have led to the development of sophisticated nanomaterials with a high encapsulation efficiency that maintain integrity until they reach their targets in vivo. Among these, mesoporous silica particles (MSP) are probably some of the most biocompatible, versatile and safe nanomaterials used in biomedical studies. MSP have been extensively explored as drug delivery carriers because of such factors as their controllable pore and nanoparticle sizes, easy surface functionalization, and high specific surface area [8,9,10,11,12,13]. As inorganic materials, silica is generally recognized to be more bio-stable than most organic compounds used in drug delivery, and this fact has given rise to the debate of the probable accumulation of SiO_2_ and possible mid–long-term toxicity [14]. However, several studies demonstrate the degradation of both mesoporous and amorphous silica upon exposure to different biological conditions. These studies establish that silica erosion and stability depend on factors such as temperature, pH, surface modifications, or the chemical composition of the medium surrounding the particle [3,5,15,16,17,18,19,20,21]. MSP have been claimed to be rapidly degraded in water [22] or physiological media [16], particularly in those containing amino-rich compounds such as cell culture medium, while serum seems to reduce degradation [3,21]. In addition, despite being a good characterization of silica dissolution in different media, there is no detailed information about silica degradation in cellulo. Given the lack of information regarding the precise sequential steps in the intracellular processing of MSP, here we investigate in detail the sub-cellular route and the interplay between the design of silica particle carriers and their degradation inside cells. 

## 2. Materials and Methods 

### 2.1. Synthesis and Characterization of Mesoporous Silica Particles 

A mixture of K_2_H_2_PO_4_ (0.857 g) and NaOH (0.145 g) in water (100 mL) was placed in a hot plate and heated up to 95 °C under magnetic stirring. Cetrimonium bromide (CTAB, 0.455 g), glycerol (15 mL), and tetraethyl orthosilicate (TEOS, 4.875 mL) were added to the mixture. The mixture was left for 2 h in agitation. After the mixture cooled down, it was centrifuged at 3300 *g* for 15 min. The pellet was resuspended first, with a mixture of EtOH/H_2_O (*v*/*v*) and later, four times more with EtOH by centrifugation/redispersion cycles (3300 *g*, 10 min). Upon synthesis, the CTAB was removed by thermal calcination at 600 °C, leaving mesopores in the particles.

A JEM 1011 equipped with a high-resolution Gatan digital camera (JEOL, Japan) was used for transmission electron microscopy (TEM) particle morphology and size characterization. From each measurement, more than 100 particles were counted to have a reliable size distribution. Zeta (ζ) potential of the particles was measured with a Zetasizer Nano-Zs, to confirm the different coatings and ethanol stability. A full characterization of the same MSP batch (including the pore size) is detailed in previous studies [23,24]. 

### 2.2. MSP Labelling and Carbon Nantoube (CNT) Coating 

Fluorescent labelling of the MSP was performed by two techniques, covalently binding rhodamine B isothiocyanate (RBITC) [25,26] or electrostatically by coating the particles with poly-l-lysine labelled with fluorescein isothiocyanate (FITC-Poly-l-l). RBITC binding to the MSP was done following the next protocol. MSP were functionalized with (3-aminopropyl) triethoxysilane (APS) adding 0.25 mL of APS in 5 mL of an ethanolic dispersion of MSP (8.7 mg/mL). After stirring 3 h, the excess of reagents was removed by centrifugation/redispersion cycles with ethanol (3300 *g*, 20 min). Then, MSP were dispersed in 10 mL of EtOH and added to 10 mL of an ethanolic solution of RBITC (0.32 mg/mL). Excess of dye was removed by three centrifugation/redispersion cycles (3300 *g*, 20 min). 

FITC-Poly-l-l coating was carried out using the “layer-by-layer” technique [27]. For this purpose, 12.5 mg of particles were added to a 25 mL solution of poly-l-lysine (0.25 mg/mL) in 0.5 M NaCl. The mixture was kept under stirring for 30 min at room temperature. The excess of the polyelectrolyte was eliminated by three cycles of centrifugation/resuspension in water (3300 *g*, 20 min).

Oxidized CNT coating was performed on poly-l-lysine coated MSP. A full characterization of the batch of nanotubes (from Nanocyl, ref: NC3100™) is available in the previous publications of the group [25,26,28]. In short, MSP were electrostatically attached to the particles. For this, oxidized CNTs resuspended in water were incubated with the particles for 12 h in mild rotation. Excess of CNT was removed by centrifugation/redispersion cycles in water (3300 *g*, 20 min). 

### 2.3. Cell Culture, Fluorescent Labelling, and Confocal Microscopy

HeLa cells were obtained from the European Molecular Biology Laboratory (EMBL) Cell Bank. NSC-34 neural cell line was kindly provided by Dr. Angelo Poletti, of the Università degli Studi di Milano. Cells were cultured under standard conditions in Eagle’s minimal essential medium (MEM) containing 10% fetal bovine serum (FBS) and antibiotics (from Gibco, Thermo Fisher Scientific, Waltham, MA, USA). Cells were incubated with 15 μg/mL of particles (unless otherwise indicated in the text), resuspended, and functionalized by mild sonication in standard tissue culture medium containing serum. The concentration of particles that was administered to the cells was based on our previous experience and on published literature that describes the biocompatibility of this nanomaterial [29,30,31,32,33,34].

To study the sub-cellular localization of particles, cells were fixed in 4% paraformaldehyde. Cultures were stained with different dyes for fluorescent confocal imaging. Nuclei and lysosomes were stained with Hoechst dye (Bisbenzimide) (from Sigma-Aldrich, St. Louis, MO, USA) and LysoTracker^®^ Deep Red (Thermo Fisher), respectively. Acridine orange (Sigma-Aldrich) was used to stain the cell contouring and vesicles limits. CellTracker™ Green CMFDA (Thermo Fisher) was used to stain one type of cell in the endocytosis–exocytosis experiments. Microtubules were immunolabeled with the anti-α-tubulin (B512) (Sigma-Aldrich) antibody that was recognized by a secondary goat anti-mouse immunoglobulin G (IgG) conjugated Alexa Fluor 647 (Molecular Probes, Thermo Fisher). Phalloidin-tetramethylrhodamine B isothiocyanate (Sigma-Aldrich) was used to stain F-actin. Fluorescent confocal microscopy images were obtained with a Nikon A1R confocal microscope and processed with the NIS-Elements Advanced Research software. All confocal images are pseudo-colored. 

### 2.4. Viability Tests 

To test viability in HeLa cells, cell death and cell proliferation blockage were measured using flow cytometry in a total of ca. 10,000 cells fixed with 4% of paraformaldehyde and stained with Hoeschst in a Becton Dickinson FACS Canto II equipment. Data were analyzed using the FACS Diva software (Becton Dickinson, BD FACSDiva 7.0). Oxidative stress was measured using the H_2_DCF-DA (Thermo Fisher) molecule, which under ROS, changes to 2′,7′-dichlorofluorescein (DCF), a fluorescent molecule. Cells treated with 100 µM of H_2_O_2_ were used as a positive control. NSC-34 cell death was analyzed using a trypan blue assay and was analyzed with the BioRad-TC10 automated cell counter. This dye typically penetrates cells with a damaged cell membrane, undergoing necrosis or late apoptosis.

### 2.5. Particle Degradation/Dissolution Studies 

For the in cellulo studies, the culture medium of cells exposed to particles during 0, 12, 48, and 120 h was collected and centrifugated at 20,000 *g* for 30 min. The preparation of the samples for the inductively-coupled plasma optical emission spectroscopy (ICP-OES) analysis was performed upon digestion of 0.5 mL of sample solution with 1 mL HCl:HF (1:3) at 120 °C during 3 h in a 5 mL teflon vessel. After neutralizing the samples, they were injected and analyzed by ICP-OES in an ICP-OES Optima 4300DV (Perkin Elmer, Waltham, MA, USA) to determine the concentration of Si species in the medium. Results represent a mean of three experimental replicas. TEM was also used to analyze MSP degradation both in cellulo and in different media. For the TEM images of cells, cell pellets of trypsinized HeLa and NSC-34 cells were fixed with 1% glutaraldehyde in 0.12 M phosphate buffer, washed in 0.12 M phosphate buffer, post-fixed in 1% buffered osmium tetroxide, dehydrated, and embedded in Araldite. Ultrathin sections of the cells were placed on copper grids and stained with lead citrate-uranyl acetate. Araldite sections (ca. 70 nm) were observed using a JEOL JEM 1011 microscope. 

In vitro particle degradation was analyzed in two media-simulating physiological conditions (PBS) and lysosomal conditions. The composition of the synthetic lysosomal medium was 25 mM MES-NaOH pH 4.5 containing 0.5 mM CaCl_2_, 1 mM MgCl_2_, and 200 mM KCl, as previously described [35]. Estimation of particle diameter was obtained through TEM images after measurement >100 particles.

### 2.6. Statistical Analysis 

A Student’s *t*-test statistical analysis was carried out with Sigma Plot 8.0 (Systat Software) to evaluate the significance of a minimum of three different replicas of each experiment. The confidence level and the total number of events included in the study are indicated for each statistical analysis. Quantitative results were expressed as mean values with their corresponding standard error bars. As indicated in the text, (*) = *p* < 0.01; (**) = *p* < 0.025; (***) = *p* < 0.005.

## 3. Results and Discussion

### 3.1. Characterization of the Particles

Figure S1 shows TEM images, sizes, and the calculated ζ potential of the MSP and CNT:MSP. The MSP core displays a diameter of ca. 500 nm with mesopores of 2.5 to 3.5 nm. Particles were coated with poly-l-l as indicated in Section 2.1. Upon polymer coating, the calculated estimated ζ potential of the MSP changed from −13.2 to +40.2 mV. The coating with oxidized CNTs is indicated in Section 2.2. Particle dosages and exposure times have been fully characterized in previous studies [25,28]. 

### 3.2. Silica Particles undergo Endocytosis-Exocytosis Cycles

It is well established in the literature that most cell types capture serum-functionalized SiO_2_ particles by receptor-mediated endocytosis [26,36,37,38,39]. Upon engulfment, particles are incorporated in the endo-lysosomal vesicular route where, among others, local enzyme activation leads to the proteolysis of their protein biocorona [40,41,42]. 

**Figure 1:**
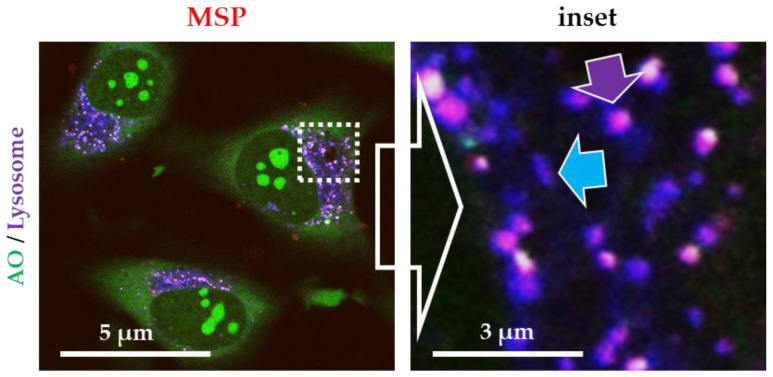
Fluorescent confocal microscopy images of live HeLa cells exposed to rhodamine B isothiocyanate (RBITC)-labelled mesoporous silica particles (MSP). Cell were stained with acridine orange (AO, green channel) and a specific marker for lysosomes (Lysotracker^®^, blue channel). All intracellular MSP were contained inside lysosomes (purple arrow). Empty lysosomes are labelled in blue (blue arrow). Free cytosolic particles (only red fluorescence) were not detected.

To verify the canonical endocytic route in our system, we exposed HeLa cells to functionalized red-silica particles (RBITC-labelled MSP). Cells were stained with endo-lysosome-specific probes (lysotracker^®^) to identify the localization of the particles. Figure 1 shows fluorescent confocal microscopy images of live cells exposed to MSP (red channel). Most endo-lysosomes display a purple color resulting from the colocalization of the red fluorescence of the particles (inside these membranes) combined with the blue fluorescence of the lysosomes (purple arrows). The quantification of more than 250 particles (localized in the endo-lysosomes or purple vesicle vs. red particles) showed that 99.7% of MSP were inside these vesicles. This result suggests that all the intracellular MSP were trapped inside endo-lysosomes, thus, confirming that serum-coated MSP follow the canonical endocytic route. 

**Figure 2:**
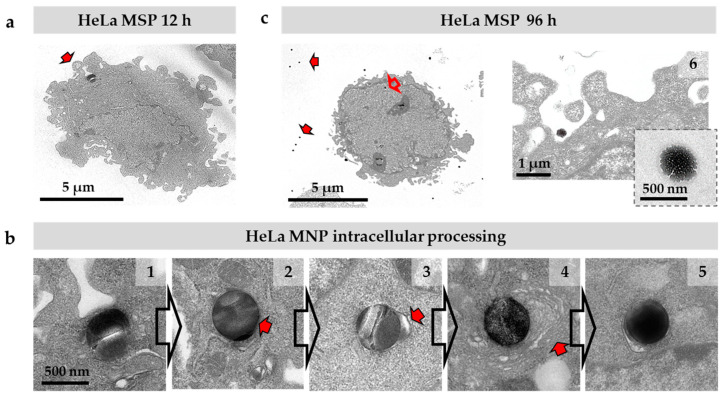
Intracellular route of MSP in HeLa cells by transmission electron microscopy images. (**a**) MSP are engulfed via endocytosis by filopodia (arrow). (**b**) Particles are immediately incorporated inside the endocytic membranes (#1 corresponds to 6 h of incubation). A thin membrane surrounding the MSP is visible in some sections (#2, arrow, corresponds to 12 h of incubation). Encapsulated particles fuse to lysosomes (#3, arrow, corresponds to 24 h of incubation). Some particles are coated with membranes (#4, arrow, corresponds to 48 h of incubation) apparently undergoing an autophagia-like process (#4, #5). After 96 h, many intracellular particles show a dense core and a compacted appearance suggesting biomolecular/biofluid infiltration (#5, corresponds to 72 h of incubation). (**c**) Many extracellular MSP are observed in cultures 96 h after the exposition (#6, inset). Extracellular particles display an eroded surface and a dense core. Cracks in the particles (#1, #2, #3) are due to artefacts resulting from ultrathin sectioning.

For further characterization of the intracellular destination of MSP, we used TEM images. HeLa cell cultures exposed to MSP during periods of up to 96 h, were fixed and processed for ultrathin sectioning (see Material and Methods section). Figure 2 shows sections of HeLa cells displaying particles at different steps of their intracellular route. MSP initially interacted with the projecting filopodia and lamellipodia of the cells that contact and capture the particles (Figure 2a, arrow). Upon endocytosis, particles are incorporated in membranous structures corresponding to endosomal vesicles (Figure 2b, #1, #2). As is typical in the receptor-mediated endo-lysosomal route, lysosomes fused to endosomes containing the engulfed MSP (Figure 2b, #3, arrow). The progressive fusion of lysosomes causes important chemical changes in the vesicular milieu resulting in acidification and the increase of reducing conditions. This activates lysosomal proteases, confining proteolysis in this intracellular membranous compartment. At this point, the proteins of the biocorona are degraded [41] and some changes in the particle’s morphology, such as core compaction, are observed, suggesting biomolecular/biofluid infiltration in the mesopores (Figure 2b, #4, #5) as described in other studies [43]. Some of these darker particles appeared surrounded by several extra layers of membranes, resembling endoplasmic reticular cisternae, pointing out an auto-phagocytic process previous to particle exocytosis (Figure 2b, #4, arrow) as suggested in previous literature [26]. Cultures exposed to particles for longer periods, i.e., 96 h, presented abundant particles free in the extracellular space (Figure 2c, red arrows). Interestingly, although we did not detect changes in the diameters of the particles along the described endo-lysosomal route (Figure 2a,b), we observed that particles in the extracellular space displayed a significantly-eroded surface and a smaller diameter compared to the original spheres, indicative of degradation in the culture medium (Figure 2c, #6, inset). 

**Figure 3:**
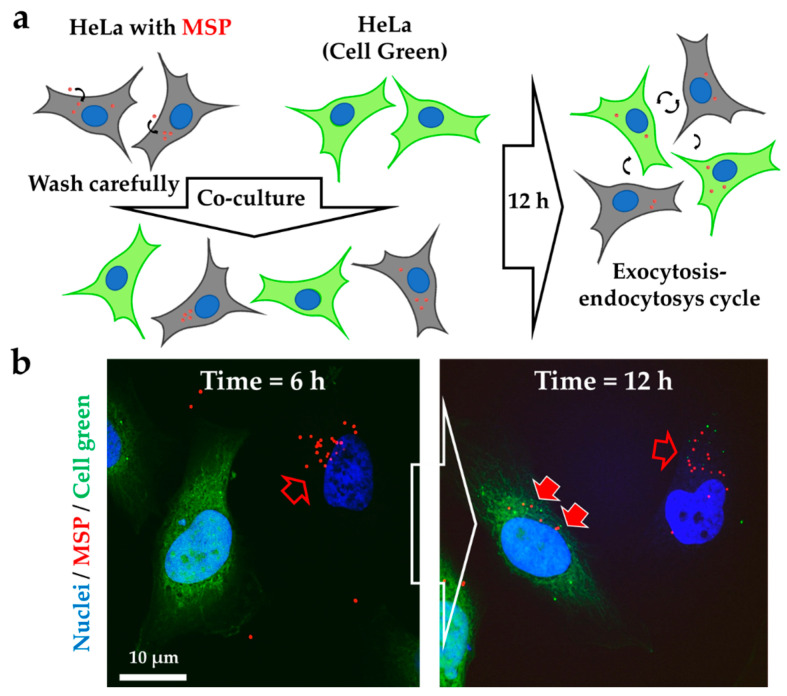
MSP undergo exocytosis–endocytosis cycles. (**a**) Experimental design. Green-labelled ‘empty’ Hela cells were co-cultured with RBITC particle-loaded HeLa cells detached and washed from a different dish (in grey) for 12 h before confocal microscopy imaging. (**b**) Projection images of co-cultured HeLa cells containing intracellular RBITC-labelled MSP (red channel, empty arrows) with green ‘empty’ cells. After 12 h, MSP were detected in the cytoplasm of the green cells (red-filled arrows) demonstrating particle exocytosis and re-endocytosis. Nuclei were stained with Hoescht (blue channel).

To investigate the exocytosis–endocytosis process, we co-cultured green “empty” HeLa cells together with unstained HeLa cells that had been previously loaded with red MSP in a different culture dish (Figure 3a). For the experiment, cells were cultured with the particles for 12 h to ensure that all MSP were endocytosed and were not attached to the extracellular membrane surface (Figure S2).

These cells were next detached from the dish, thoroughly washed to ensure no particles were transferred extracellularly, and then co-cultured with green “empty” cells. Figure 3b shows how, after 6 h of co-culture condition, although most of the particles are still inside the unstained cells, some of them have already been exocytosed. After 12 h of co-culture conditions, the originally-“empty” cells contained intracellular red particles (red solid arrows). These results, together with those shown in Figure 2, prove that MSP undergo endocytosis- exocytosis cycles as also reported by other authors [26,44,45,46].

### 3.3. Silica Particles undergo Dissolution in the Cytosol

To understand MSP degradation in the cellular realm, we used a coating system that allowed particle escape from endo-lysosomes to the cytosol. Between the few currently-available systems, we decided to coat the particles with oxidized CNT. This type of nanotubes is innocuous when attached to silica particles [25,26], while significantly increasing the interaction of the nanomaterial with the membrane of the endo-lysosomes [26]. The intimate interaction between the CNT coating and the endosome tears the vesicular membranes apart, releasing the silica particles into the cytoplasm. 

**Figure 4:**
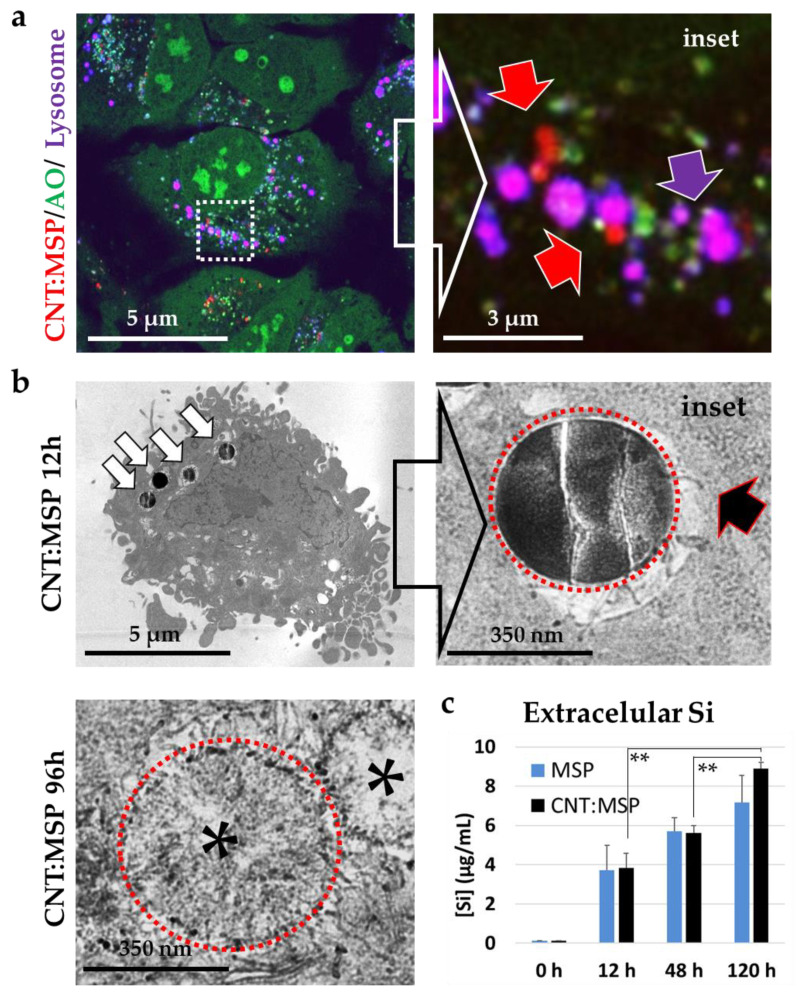
Subcellular localization and degradation of MSP with CNT coating (CNT:MSP) in HeLa cells. (**a**) Confocal microscopy images of live cells exposed to RBTIC-labelled CNT:MSP (red channel), Cells were stained with AO (green channel) and the lysosomal probe Lysotracker^®^ (blue channel). (inset) Purple arrows point to lysosomes containing particles, red arrows point to particles released into the cytosol. (**b**) TEM images of ultrathin sections of HeLa cells exposed to CNT:MSP. White arrows point to endocytosed particles. The inset shows CNTs piercing the lysosomal membranes (black arrow). Cytoplasmic CNT:MSP were significantly degraded 96 h after endocytosis, displaying empty cores (asterisks). For identification purposes, particles are surrounded by a red circle. (**c**) Quantitative ICP-OES analysis of Si in the extracellular culture media of the cells exposed to MSP or CNT:MSP during 0, 12, 48, and 120 h. The histogram represents the results average measurements of three experiments. The increment of the Si species in the culture media suggests the extracellular presence of the SiO_2_ degradation products. Results confirm extracellular MSP and intracellular CNT:MSP dissolution (** = *p* < 0.025).

MSP coated with oxidized CNT (herein CNT:MSP) were added to the cells following an identical protocol to that in Figure 1. In this case, confocal microscopy imaging revealed that red CNT:MSP appeared both inside lysosomes (Figure 4a, inset, purple arrows), and also free in the cytosol (red arrows). After 48 h exposure to the particles, 56.5% of the particles have escaped from the endo-lysosomal membranes into the cytosol (*n* > 250, Figure S3). Interestingly, 72 h after the administration of CNT:MSP, most intracellular particles had lost their RBITC fluorescent coating, suggesting cytosolic particle surface erosion (Figure S4).

TEM images of ultrathin sections of HeLa cells exposed to CNT:MSP were used to confirm that a large number of these particles were localized in the cytosol devoid of membranes. These images showed that CNT:MSP were also captured by endocytosis (Figure 4b, white arrows). But, contrary to ‘naked’ MSP, the CNT coating pierced and tore the endo-lysosomal membranes apart (Figure 4b black arrow) releasing the particles into the cytosol. TEM images also revealed a patent cytosolic silica dissolution process that was very obvious 96 h after the administration. At this time point, CNT:MSP presented a significantly-damaged and highly-porous structure, with an almost completely disappeared core (Figure 4b, asterisks). These findings suggest that the intracellular extra-vesicular cytoplasmic conditions triggered silica particle dissolution, being more corrosive than the intra-vesicular acidic and reducing media. Thus, suggesting these nanocarriers could be good candidates to preserve cargo to be delivered into the intracellular milieu.

Finally, to verify and quantify silica particle dissolution from the chemical point of view, we measured the rise of free silicon species in the cell culture media at different exposure using ICP-OES (see The Materials and Methods section) [47,48]. To eliminate putative silica fragments from the media and to uniquely quantify the dissolved silica, we centrifuged the culture samples at 20,000 *g* for 30 min. This centrifugation step guarantees that whole particles or their fragments are not included in the measurements. This way, any Si species in the culture medium can only be indicative of particle dissolution thus, the actual degradation of particles (i.e., including fragmented silica) is higher than that detected by ICP. Figure 4c shows the quantitative analysis of the measurements of the extracellular Si content after the administration of both MSP and CNT:MSP. The progressive increment of this element in the media is indicative of particle dissolution and release of the resulting silicon-free species into the surrounding media, as previously discussed [15,16,19,21,22,49]. Results obtained for MSP’s and CNT:MSP degradation do not show statistically significant differences. This can be explained because MSP are dissolved extracellularly thus raising the Si content in the media. Both, the extracellular and the intracellular media are very similar in pH and salts, which makes the degradation time in both types of particles similar. 

### 3.4. MSP Degradation In Vitro Recapitulates the In Cellulo Results

Archive literature suggests silica needs to be enzymatically degraded in eukaryote cells by specific enzymes such as silicateins or silases as it is the case in organisms such as marine sponges [50]. Thus, the steps in the intracellular degradation of the particles and the effect of the salt and pH in the process were next studied using buffers mimicking the chemical conditions of the cytosol (or extracellular media) and the lysosomes. Silica particles were incubated 1 to 7 days at 37 °C in mild agitation, and degradation was evaluated using TEM images of the particles (see Material and Methods section). 

**Figure 5:**
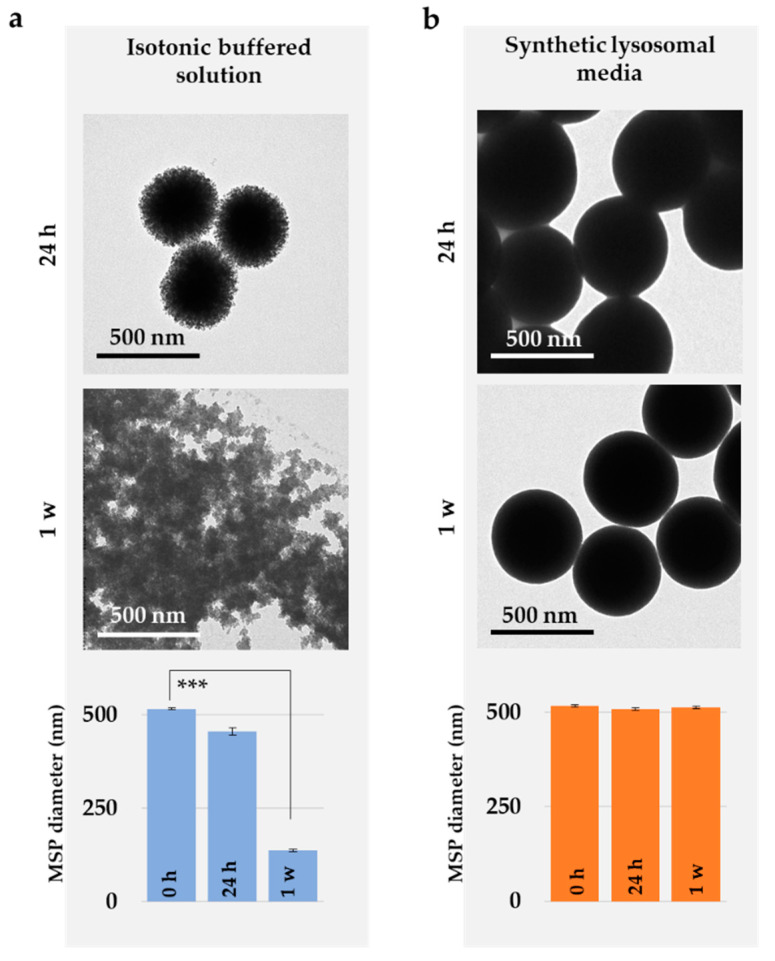
Degradation of MSP in synthetic biomimetic solutions. Representative TEM images of MSP after incubation for 24 h and 1 week in (**a**) isotonic buffered solution mimicking the intra/extracellular milieu (PBS) or (**b**) synthetic lysosomal media. Statistical evaluation of the size change in particle diameter upon incubation in media (*** p < 0.005). Control particles are shown in Figure S1.

Figure 5 demonstrates how buffers mimicking the composition of the cytoplasmic milieu or extracellular media triggered a fast MSP degradation compared to the synthetic lysosomal media. Hence these results reinforce the idea that MSP degradation is accelerated in isotonic buffers (pH 7.4, 137 mM NaCl) mimicking the chemical conditions of the cytosol, while in lysosomal buffers, MSP displayed no apparent changes for at least 7 days (Figure 5b) as reported in previous studies [3,49,51]. These results strongly suggest that MSP must be primarily considered as hydrolytically degraded and not biodegraded [48].

### 3.5. Silica Dissolution is Innocuous to Epithelial Cells

**Figure 6:**
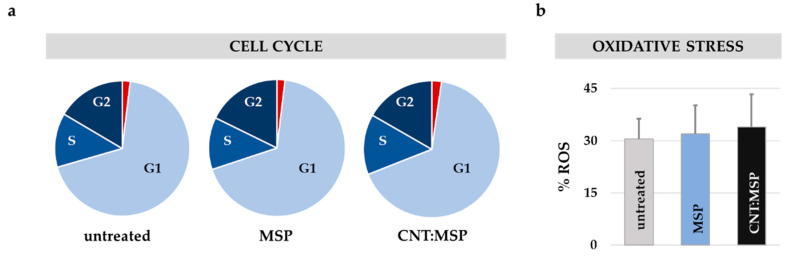
Silica dissolution is innocuous to HeLa cells. (**a**) HeLa cell cycle and viability 72 h after exposure to MSP or CNT:MSP. Cell cycle stages (in blue shades, G1 = non-dividing; S = duplicating phase; G2 premitotic/mitotic phase); and apoptotic cell (red) percentages in cultures of untreated (control) cells or cultures exposed to MSP or CNT:MSP. (**b**) Analysis of the oxidative stress in the cultures. In HeLa cells, no statistically-significant rise in the % of ROS was observed upon administration of MSP or CNT:MSP compared to untreated controls. The positive control (corresponding to 100% of ROS) was done adding 100 μM of H_2_O_2_.

Live-cell studies with MSP did not reveal any detectable cytological finding indicative of toxicity (Figure 1, Figure 2, Figure 3 and Figure 4). This issue was confirmed by flow cytometry examining cell death (apoptosis) and possible changes in the cell cycle. This technique allows measuring approximately 10,000 events (cells) per condition simultaneously providing a good statistical evaluation of the cytotoxicity. The results shown in Figure 6 demonstrate how cells exposed to 15 μg/mL of MSP or CNT:MSP during 72 h, displayed no significant signs of cell death (in red) or alterations in the mitotic cycle compared to untreated control cells. 

The viability study was complemented with the quantification of the cellular oxidative stress (reactive oxygen species, ROS) at the same exposure times. No statistically-significant changes were observed in MSP- or CNT:MSP-treated cells compared to untreated controls (Figure 6b). These results lead us to conclude that, at the concentration used, intracellular silica particle degradation is innocuous to HeLa cells.

### 3.6. Intracellular Silica Dissolution is Innocuous to Motor Neurons 

Motor neuron diseases are disorders that affect selectively to the neurons that control voluntary muscles of the body. These cells have a very active metabolism and have to last a lifetime without replacement. Hence, these cells are very vulnerable to chemical or metabolic insults in the medium–long term. 

**Figure 7:**
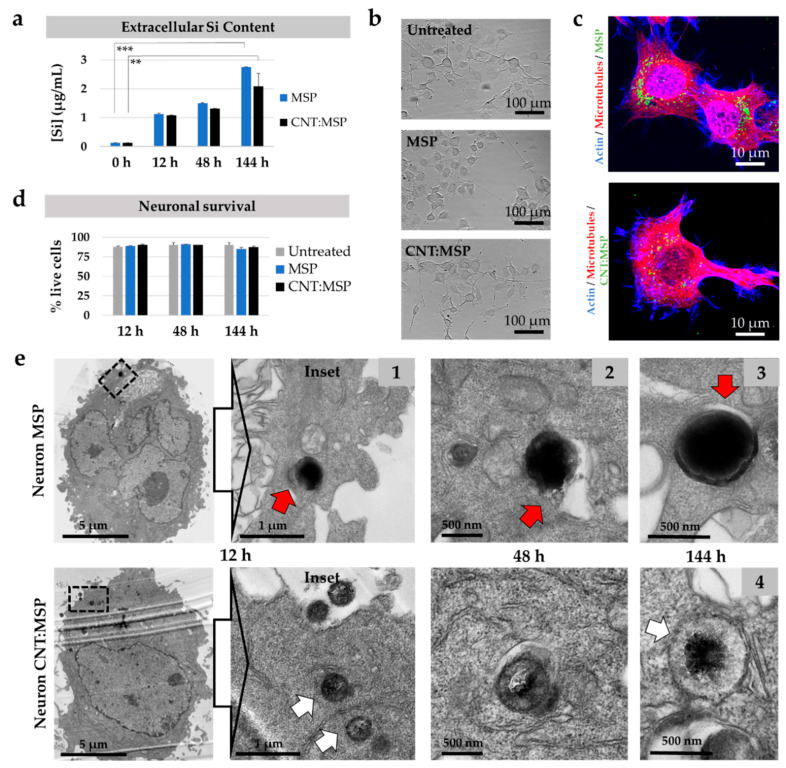
Intracellular route and degradation of MSP in neurons. (**a**) Quantitative ICP-OES analysis of extracellular Si in cultures of NSC-34 neurons exposed to MSP and CNT:MSP. The rise of the Si species corroborates particle dissolution in these cells at the indicated times (** *p* < 0.025, *** *p* < 0.005). (**b**) Phase-contrast images of control neuronal cultures and cells exposed to 15 µg/mL of MSP and CNT:MSP for 6 days. No deleterious morphological changes were observed. (**c**) Fluorescent confocal images of neurons displaying MSP and CNT:MSP (green channel). No abnormalities in the cytoskeleton of microtubules (red channel) or actin (blue channels) were detected. (**d**) Neuronal survival upon exposure to the two types of particles was not affected. (**e**) TEM images of ultrathin sections of motor neurons containing intracellular MSP (top) and CNT:MSP (bottom). Particles reproduce the same destinies and degradation patterns described for epithelial cells. Red arrow points at the endosomal membranes surrounding a particle. White arrow shows the eroded surface of a cytosolic CNT:MSP.

Until now, there have been no safe vectors that can be used to deliver therapeutic agents (drugs, repair genes or proteins, or RNAs, etc.) to motor neurons. Given the excellent biocompatibility observed for the MSP in epithelial cells, we considered that MSP could be ideal nanocarriers in neural repair and thus, we investigated the intracellular degradation of these particles in the cytoplasm of neurons. For this purpose, we exposed NSC-34 cells (see Material and Methods section) to both types of MSP following the protocols described for HeLa cells. The results, summarized in Figure 7, show how motor neurons can efficiently dissolve silica particles (Figure 7a), and, despite previous reports [52,53], our tests show the innocuity of MSP in these cells. Neurons displayed no morphological changes indicative of degeneration, even after 6 days of exposure to MSP (Figure 7b,e). The healthy-looking appearance of the cells in culture (Figure 7b,c,e) and the statistical analysis of neuronal survival after 6 days of exposure to both types of particles (Figure 7d) showed undetectable toxicity in treated neurons. Finally, TEM images of ultrathin sections of the motor neurons (Figure 7e) confirmed that both MSP and CNT:MSP followed virtually identical intracellular destinies in these cells to HeLa. This fact supports the hypothesis that most eukaryotic cells are likely to dissolve silica particles efficiently and safely.

## 4. Conclusions

This work demonstrates how silica particles, contrary to what is usually expected for most nanomaterials, degrade primarily in the cytoplasmic realm or extracellularly, but not in lysosomes. The results obtained also imply that this phenomenon requires chemical dissolution rather than biochemical (enzyme-assisted) events, meaning that any cell or even the extracellular milieu can trigger silica nanoparticle degradation. We also showed how through triggering endo-lysosomal escape, MSP intracellular degradation is enhanced. These studies provide clues to how controlling the spatiotemporal dissolution of MSP nanocarriers can serve to protect lysosome-sensitive therapeutic compounds during the endo-lysosomal transit to the cytoplasm, thus preventing the release of therapeutic compounds until cytosolic arrival. Furthermore, innocuous silica dissolution events occur, not only in epithelial cells, but also in motor neurons, and probably in most cell types. This fact could help in the development of neuronal-directed safe nanocarriers for the treatment of neurodegenerative disorders such as motor neuron diseases, uncommon conditions that affect the brain and nerves, nearly always fatal, that have no treatment to prevent, alter, or cure the disease. 

## Supplementary Materials

The following are available online at https://www.mdpi.com/1999-4923/12/6/487/s1, Figure S1: Characterization of the silica particles, Figure S2: Fluorescent confocal microscopy image of HeLa cells after 12 h of the administration of MSP, Figure S3: CNT:MSP escape from endo-lysosomes, Figure S4: Cytosolic particle surface degradation.
