# Supplementary material for: Engineering Sub-Cellular Targeting Strategies to Enhance Safe Cytosolic Silica Particle Dissolution in Cells
Source: Pharmaceutics. 2020 May 28;12(6):487. doi: 10.3390/pharmaceutics12060487 (PMC7356774; doi:10.3390/pharmaceutics12060487)
Supplement: Supplementary file 1 [file pharmaceutics-12-00487-s001.pdf]

pharmaceutics

MDPI

Supplementary Materials: Engineering Sub-Cellular Targeting Strategies to Enhance Safe Cytosolic Silica Particle Dissolution in Cells

Nerea Iturrioz-Rodríguez, Miguel Ángel Correa-Duarte, Rafael Valiente and Mónica L. Fanarraga

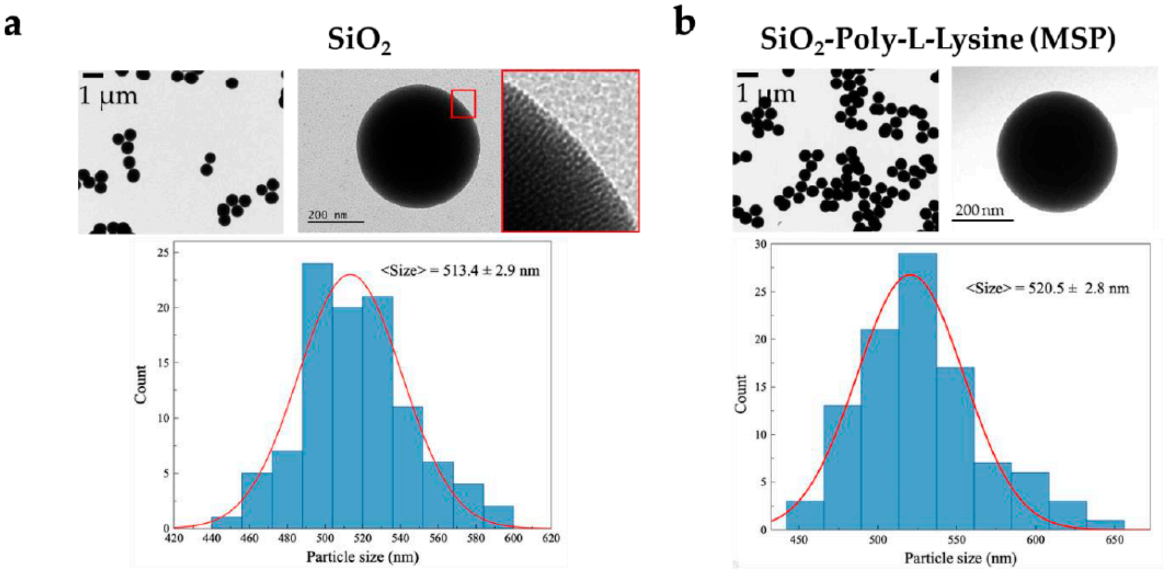

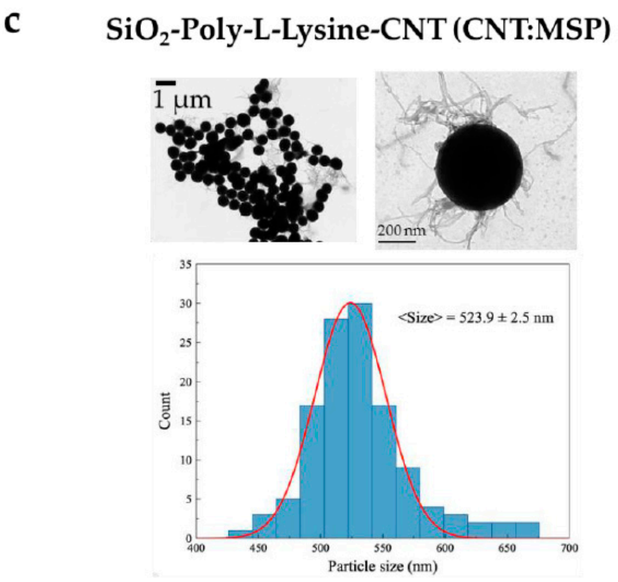

d

In ethanol	ζ potential (mV)
SiO_2	- 13.2 (± 0.3)
MSP	+ 40.2 (± 1.1)
CNT	- 33.4 (± 2.6)
CNT:MSP	+ 6.8 (± 0.8)

Figure S1. Characterization of the silica particles. (**a**) TEM images and size distribution of silica particles, the amplication of the image in red shows the pore structure of the particles. (**b**) TEM images and size distribution of silica particles with the Poly-L-Lysine coating (MSP), (**c**) TEM images and size distribution of MSP with the CNT coating. (**d**) Calculated ζ potential of the three types of particles and the carbon nanotubes.

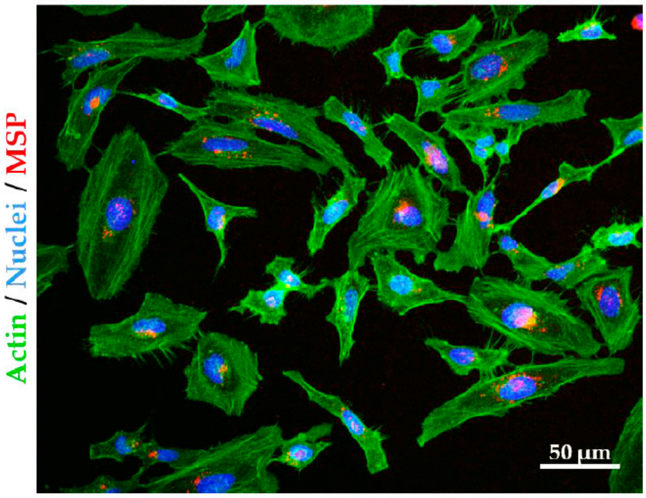

Figure S2. Fluorescent confocal microscopy image of HeLa cells after 12 h of the administration of MSP. At this time point most particles (red channel) are already in the cytoplasm of the cells.

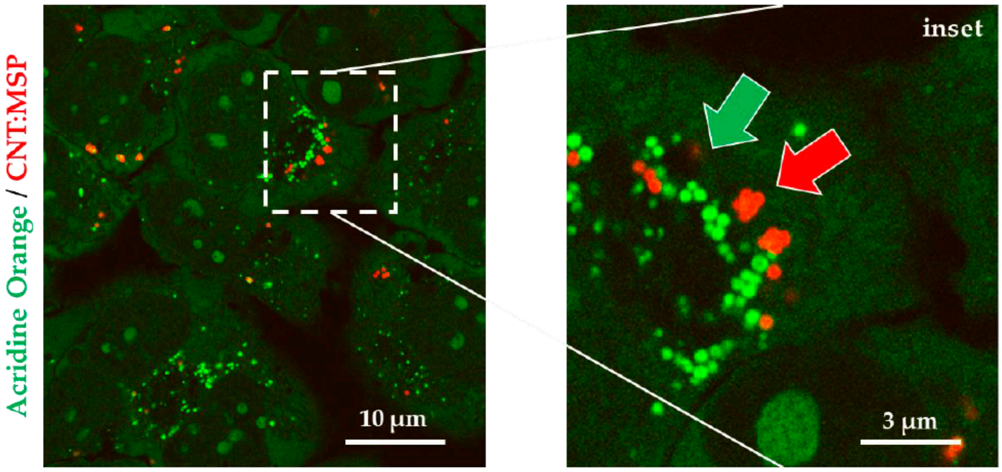

Figure S3. CNT:MSP escape from endo-lysosomes. Fluorescent confocal projection images of HeLa cells exposed to RBITC-labelled CNT:MSP (red arrow) during 48 h. Many particles have escaped into the cytoplasm and do not co-localize with the vesicles (green channel, green arrow).

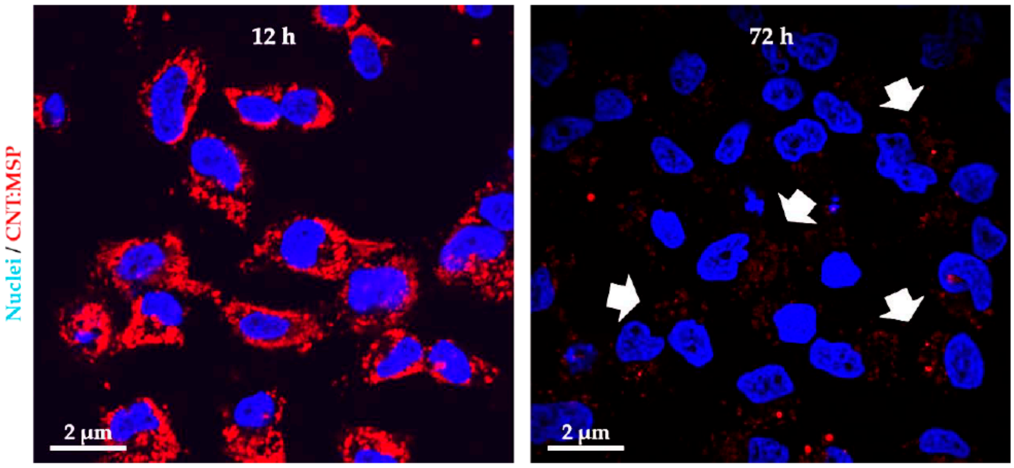

Figure S4. Cytosolic particle surface degradation. Fluorescent confocal projection images of HeLa cells exposed to RBITC-labelled CNT:MSP. After 72 h the surface RBITC-coating of the particles (red channel) has virtually disappeared suggesting surface erosion (white arrows).
